# Comparative Analysis of Thin and Thick MoTe_2_ Photodetectors: Implications for Next-Generation Optoelectronics

**DOI:** 10.3390/nano14221804

**Published:** 2024-11-11

**Authors:** Saddam Hussain, Shaoguang Zhao, Qiman Zhang, Li Tao

**Affiliations:** Center for Quantum Physics, Key Laboratory of Advanced Optoelectronic Quantum Architecture and Measurement (MOE), School of Physics, and Center for Interdisciplinary Science of Optical Quantum and NEMS Integration, Beijing Institute of Technology, Beijing 100081, China; 3820202051@bit.edu.cn (S.H.); zhaoshaoguang@bit.edu.cn (S.Z.); zqm@bit.edu.cn (Q.Z.)

**Keywords:** MoTe_2_, photodetectors, optoelectronic performance, thin films, thick films, responsivity

## Abstract

Due to its outstanding optical and electronic properties, molybdenum ditelluride (MoTe_2_) has become a highly regarded material for next-generation optoelectronics. This study presents a comprehensive, comparative analysis of thin (8 nm) and thick (30 nm) MoTe_2_-based photodetectors to elucidate the impact of thickness on device performance. A few layers of MoTe_2_ were exfoliated on a silicon dioxide (SiO_2_) dielectric substrate, and electrical contacts were constructed via EBL and thermal evaporation. The thin MoTe_2_-based device presented a maximum photoresponsivity of 1.2 A/W and detectivity of 4.32 × 10^8^ Jones, compared to 1.0 A/W and 3.6 × 10^8^ Jones for the thick MoTe_2_ device at 520 nm. Moreover, at 1064 nm, the thick MoTe_2_ device outperformed the thin device with a responsivity of 8.8 A/W and specific detectivity of 3.19 × 10^9^ Jones. Both devices demonstrated n-type behavior, with linear output curves representing decent ohmic contact amongst the MoTe_2_ and Au/Cr electrodes. The enhanced performance of the thin MoTe_2_ device at 520 nm is attributed to improved carrier dynamics resulting from effective electric field penetration. In comparison, the superior performance of the thick device at 1064 nm is due to sufficient absorption in the near-infrared range. These findings highlight the importance of thickness control in designing high-performance MoTe_2_-based photodetectors and position MoTe_2_ as a highly suitable material for next-generation optoelectronics.

## 1. Introduction

The growth of innovative photodetectors remains critical for diverse applications, including imaging, optical communication, environmental monitoring, and biomedical sensing [1]. Two-dimensional materials, particularly transition metal dichalcogenides (TMDs), have become capable contenders for next-generation photodetection technologies due to their outstanding optical, electrical, and mechanical characteristics [2]. Among these materials, molybdenum ditelluride (MoTe_2_) has expanded significantly due to its adjustable band gap, excellent electron mobility, and excellent photoresponsivity, making it ideal for electronic and optoelectronic devices [3]. MoTe_2_ is classified into three distinct structural phases: the semiconducting trigonal-prismatic 2H- or α-phase, the semi-metallic and monoclinic 1T′- or β-phase, and the semi-metallic orthorhombic γ-structure, which allows for diverse expedient configurations and functionalities. The phase tunability and variable thickness-dependent possessions deliver prospects for improving device performance across a broad spectrum of applications [4]. The thickness of MoTe_2_ layers significantly affects their optoelectronic characteristics, with thickness-dependent layers often exhibiting superior photoresponse, faster switching times, and enhanced charge carrier dynamics. The survey of contemporary research has merged several schemes to enhance the efficiency of MoTe_2_-based photodetectors. In the end, the authors proved that the enhancement of the photocurrent response sensitivity or detectivity of MoTe_2_ photodetectors was achieved by incorporating all-dielectric TiO_2_ metalenses. These metalenses enhanced the use of photons in the operation of the device to improve results [5]. However, their attention mainly focused on exploring other external optical architectures to improve the performance, but they did not investigate how the thickness changes the photoresponse of MoTe_2_. In another work, a MoTe_2_ waveguide photodetector with a few layers was still under consideration; it offered reliable performance in near-infrared (NIR) regions, mainly for high-speed optical data communication [6]. Although this research focused on exceeding the external quantum efficiency of MoTe_2_ for NIR applications, it failed to determine the effect of varying material thickness on its efficiency in both the visible and NIR regions. The contribution of our work is made in the comprehensive comparative study of thin (8 nm) and thick (30 nm) MoTe_2_ layers regarding the dependence of the device performance on the layer thickness in the visible (520 nm) and near-infrared (1064 nm) regions. In contrast to previous studies, which mainly looked at external modifications or specific wavelength use of MoTe_2_, this study systematically investigates the thickness-dependent characteristics of the material. We also show that a few thinner MoTe_2_ layers have better device performance at the visible range because of the improved electric field penetration. Conversely, the absorption of light in the thicker layers is superior in the NIR range. It gives significant information about the relationship between the thickness of MoTe_2_ and how it can be tuned for different photodetection applications, making it possible to have a different perspective on tuning the MoTe_2_ photodetectors. The work presented herein is also novel due to the absence of comparative analysis research focusing on the thickness of the MoTe_2_ photodetector. It surpasses prior research by offering specific recommendations for thickness optimization in future device designs. Recent studies have shown that thin MoTe_2_ photodetectors demonstrate improved performance metrics, such as higher responsivity, specific detectivity, and faster response times, than thicker layers. These performance improvements are primarily attributed to the higher surface-to-volume ratio, better electric field penetration, and enhanced charge transport properties in thinner layers, facilitating more efficient photogenerated carrier extraction. As a result, optimizing the thickness of MoTe_2_ layers becomes a critical parameter in the design and development of high-performance photodetectors [7].

Despite these promising characteristics, systematic studies are still needed on the comparability performance of MoTe_2_ photodetectors with varying thicknesses under different operating conditions. Understanding the impact of thickness on critical parameters, for instance, responsivity, detectivity, and response time, is essential for designing and optimizing high-performance devices. Our study comprehensively analyzes thin (8 nm) and thick (30 nm) MoTe_2_-based photodetectors fabricated with Au and Cr electrodes on a silicon substrate. We systematically explore the optoelectronic properties of these devices, focusing on their electrical and photoresponse characteristics under dark and illuminated conditions.

Our findings disclose that thin MoTe_2_-based photodetectors show superior performance in areas such as responsivity, specific detectivity, and external quantum efficiency, which were 1.2 A/W, 4.32 × 10^8^ Jones, and 285%, respectively, compared to thicker MoTe_2_ layers, which were 1.0 A/W, 3.6 × 10^8^ Jones, and 238% at 520 nm, respectively. Similarly, thin photodetectors demonstrated responsivity of 1.1 A/W, specific detectivity of 3.96 × 10^8^ Jones, and EQE of 127%, compared to thick-based MoTe_2_, which had photo responsivity of 8.8 A/W, detectivity of 3.19 × 10^9^ Jones, and EQE value of 1027% at 1064 nm. Specifically, the enhanced performance of thin MoTe_2_ layers is due to improved carrier dynamics, which result from the effective penetration of the electric field into the material, leading to better charge separation and reduced recombination rates at 520 nm. The improved performance of thick-based MoTe_2_ at 1064 nm is because MoTe_2_ has sufficient absorption in the (NIR) range because of its reduced energy gap. This study emphasizes the position of exact thickness switches in fabricating MoTe_2_-based photodetectors. This performance gap emphasizes the critical impact of material thickness on device efficiency, making it a crucial feature in the engineering of photodetectors for optoelectronic applications. By strategically manipulating the thickness of MoTe_2_, it is possible to tailor its photoresponse properties toward exact application ratios, paving the way for developing next-generation photodetection technologies. This work highlights the potential of thin and thick MoTe_2_ photodetectors for high-performance photodetectors and provides valuable insights into the design principles that govern the performance of 2D material-based devices. Our comparative study of thin and thick MoTe_2_ photodetectors establishes a foundation for future research to optimize TMD-based devices by strategically manipulating material thickness and heterostructure design.

## 2. Materials and Methods

The fabrication process for thick- and thin-based MoTe_2_ devices begins with the exfoliation of MoTe_2_ from a 2H phase provided by Nanjing MKNANO Tech. Co., Ltd., Nanjing, China (www.mukenano.com) (accessed on 15 September 2024). These exfoliated films are, at that moment, carefully positioned on Si, covered by a 285 nm thick SiO_2_ film as the dielectric foundation. AFM is employed to conduct a thorough structural examination, determining the precise thickness and configuration of the MoTe_2_ sheets. Following this, electrode patterns are created using electron beam lithography (EBL). The final step involves thermal evaporation to deposit a 5 nm chromium (Cr) base layer and a 50 nm gold (Au) upper layer, forming thick and thin MoTe_2_-based devices. The thickness and morphology of various MoTe_2_ layers were examined using atomic force microscopy (Bruker Multi-Mode 8, Bruker Corporation, Karlsruhe, Germany). Raman spectroscopy analysis (WITec alpha 300R, Oxford Instruments plc, Wiesbaden, Germany) is taken to confirm the thick and thin MoTe_2_ structure at an excitation wavelength of 532 nm. A probe stage with a Keithley 2636b semiconductor device analyzer was used to perform electrical measurements on the devices.

## 3. Results and Discussion

### 3.1. Structural Features of Thin and Thick MoTe_2_

**Figure 1:**
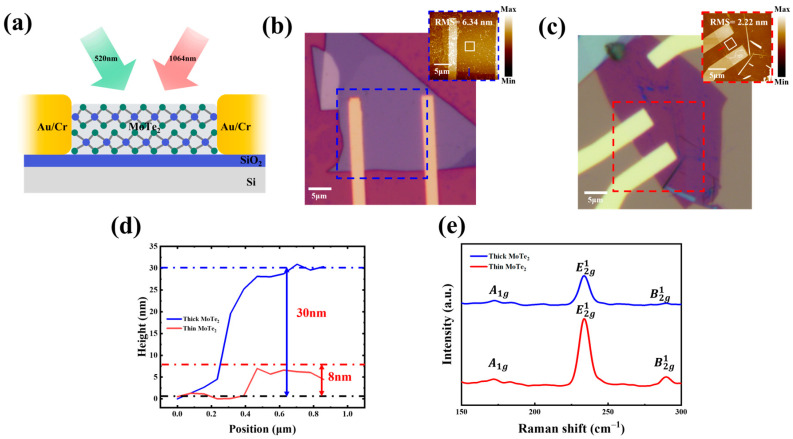
(**a**) Schematic of the MoTe_2_-based photodetector apparatus exposed to light at 520 and 1064 nm. The device comprises MoTe_2_ layers on top of a SiO_2_/Si substrate coupled with Au/Cr electrodes. (**b**) Microscopic view of a thick MoTe_2_ photodetector through Au/Cr electrodes. (**c**) Microscopic view of thin MoTe_2_ device through Au and Cr electrodes. (**d**) AFM thickness profile depicting thick and thin MoTe_2_ flakes, corresponding to inset in (**b**,**c**), with RMS of 6.34 nm and 2.32 nm, respectively. (**e**) Raman spectra of thick and thin MoTe_2_ flakes on SiO_2_/Si substrate for the excitation wavelengths of 532 nm.

The fabrication process of thin and thick MoTe_2_ heterostructure layers commenced with the exact creation of several layers of 2H-phase MoTe_2_ onto Si substrate, featuring 285 nm dielectric made from SiO_2_, serving as the dielectric layer. The device’s electrodes were fabricated using EBL, tracked by the thermal evaporation of Cr and Au films with 5 nm and 50 nm thicknesses, respectively. Figure 1a illustrates the schematic of the MoTe_2_ device with two electrodes made of Au and Cr on a SiO_2_/Si substrate.

Figure 1b,c shows a photographic and inset AFM image of a thick and thin MoTe_2_ photodetector device with Au and Cr electrodes. As indicated in Figure 1d, the thickness of the MoTe_2_ nanosheet channel is approximately 8 nm and 30 nm. This suggests that the thin MoTe_2_ comprises a few layers, while the thick MoTe_2_ is bulky. The thickness root means square (RMS) values of thick and thin MoTe_2_ devices are 6.34 and 2.32 nm, respectively, which confirm that thick surface morphology has greater roughness or variability in its properties compared to thin-based MoTe_2_, whose surface is flat, as shown in the inset of Figure 1b,c.

Figure 1e shows the Raman of thick and thin MoTe_2_, respectively. Raman shows the changes in responses of both thick and thin MoTe_2_ devices for excitation at 532 nm. It shows that three distinctive peaks are observed: the in-plane E^1^_2g_ mode (~232 cm^−1^), the out-of-plane A_1g_ mode (~171 cm^−1^), and the bulk in-active B^1^_2g_ phonon mode (~288 cm^−1^). This latter peak is absent in monolayers, which easily enables their identification for a few layers, as shown in the figure. It confirmed a few layers for thin MoTe_2_. Furthermore, in the case of thick MoTe_2_, the positions of A_1g_, E^1^_2g_, and B^1^_2g_ are shifted, with the intensity of E^1^_2g_ and B^1^_2g_ decreasing, and A_1g_ shows a weak signal, which confirms that MoTe_2_ is in bulk nature. The sharp, intense peak for the thin layer and the broader, more subdued features for the thick layer align well with known behaviors of MoTe_2_ as influenced by its thickness. The result is confirmed in the literature [8].

### 3.2. Electrical Properties

**Figure 2:**
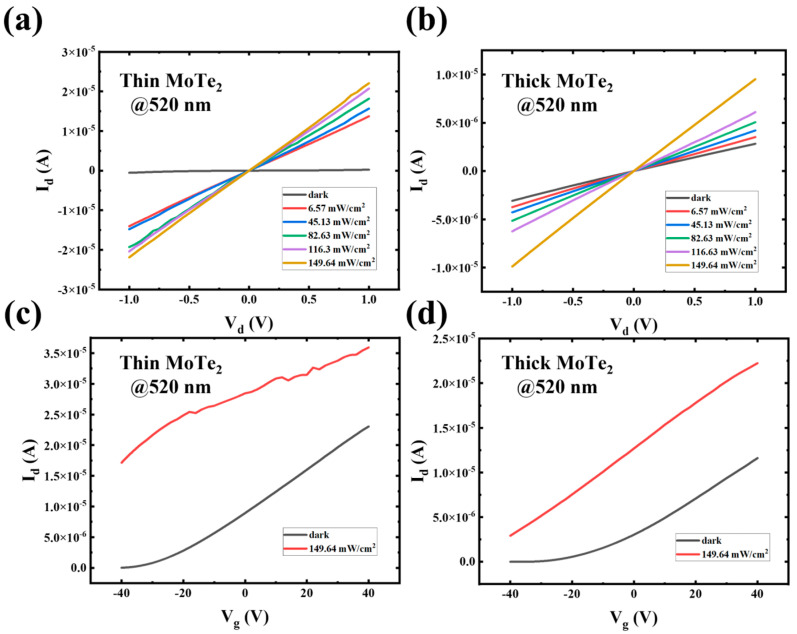
Electrical performance of thin MoTe_2_ and thick MoTe_2_-based photodetector. (**a**) Output characteristic curves of thin-based MoTe_2_ devices under a wavelength of 520 nm with varying illumination power densities spanning from 6.57 mW/cm^2^ to 149.64 mW/cm^2^. (**b**) Output characteristic curves of thick-based MoTe_2_ device under a wavelength of 520 nm with varying illumination power densities spanning from 6.57 mW/cm^2^ to 149.64 mW/cm^2^. (**c**) Transfer characteristic curves of thin-based MoTe_2_ device under dark and light power density of 149.64 mW/cm^2^ (*V*_d_ = +1 V). (**d**) Transfer characteristic curves of thick-based MoTe_2_ device under dark and light power density of 149.64 mW/cm^2^ (*V*_d_ = +1 V).

The output and transfer characteristics were also obtained under different illumination powers, as shown in Figure 2a–d. It indicates a rise in photocurrent with increasing illumination power. The surge in photocurrent with increasing light intensity suggests that the photodetector demonstrates a significant photoconductive effect, which is common in semiconductor materials. The linearity of *I*_d_*-V*_d_ characteristic curves shows an outstanding ohmic contact among the MoTe_2_ to the Cr electrodes [9]. When exposed to light, the increased current under illumination is due to the production of electron-hole pairs in the MoTe_2_ thin film. As the light intensity increases, more charge carriers are created, leading to higher currents. However, the photocurrent variation in a thin-based MoTe_2_ photodetector is more significant than that of a thick-based photodetector. This could be due to better light absorption and charge transport properties in the thinner MoTe_2_ layer, which can trap and generate more charge carriers than thicker layers. Under dark conditions, the current remains near zero, confirming a low dark current compared to thick-based MoTe_2_, as illustrated in Figure 2a–c, which displays the transfer characteristics (*I*_d_ versus gate voltage *V*_g_) of the thin MoTe_2_ device under dark conditions and illumination (149.64 mW/cm^2^). Under illumination, the photocurrent increases significantly across the entire range of gate voltages compared to the dark condition, which shows n-type semiconductors. This increase in photocurrent with light results from the photoexcitation of electrons, contributing to the rise in the total current flowing through the device. Similarly, Figure 2c,d shows the transfer characteristics for the thick MoTe_2_ device under dark and illuminated conditions, indicating that drain current increases with changing *V*_g_, proving that thick-based MoTe_2_ is an n-type semiconductor. As in the thick device, the thin MoTe_2_ device shows an increase in photocurrent under light compared to dark conditions. However, there is slight nonlinearity in the drain current of thin-based MoTe_2_ as light illumination increases, which is because there should be little trap in the thin MoTe_2_ layer or interface between MoTe_2_ and gate dielectric. However, the overall current values under both dark and light conditions are lower in the thick device compared to the thin one at 520 nm. This suggests lower light absorption and fewer photo-generated carriers in the thick MoTe_2_ layer. The thin MoTe_2_-based photodetector outperforms the thick MoTe_2_ device in terms of photocurrent generation and overall responsiveness to light. The elevated ratio of surface area to volume enhances light absorption and charge transport at 520 nm. It reduces the influence of surface states, making the thin MoTe_2_ device more suitable for applications requiring high photocurrent under light exposure.

**Figure 3:**
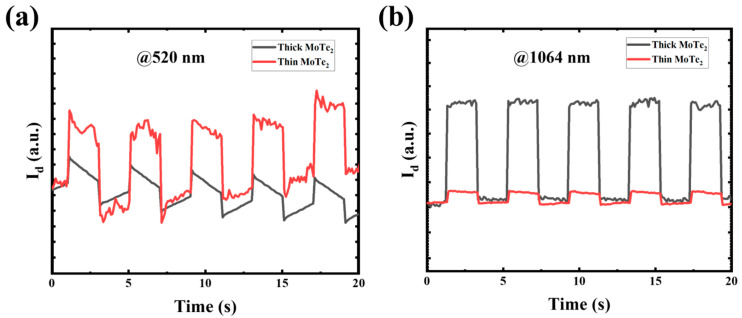
Photoresponses of thick and thin MoTe_2-_based devices. (**a**) Time-dependent photoresponses of thick and thin MoTe_2_ at *V*_d_ = +1 V and *V*_g_ = 0 V under 520 nm light illumination. (**b**) Time-dependent photoresponses of thick and thin MoTe_2_ at *V*_d_ = +1 V and *V*_g_ = 0 V under 1064 nm light illumination.

Figure 3a,b presents the time-dependent photoresponse of the devices operating at *V*_d_ of +1 V with periodic light on/off switching. The devices demonstrate a stable and repeatable photocurrent response across the 520 to 1064 nm wavelength range. The photoresponse of thin-based MoTe_2_ is improved compared to thick-based MoTe_2_ devices at 520 nm, as shown in Figure 3a. This is because MoTe_2_ is thinned down to a few atomic layers (monolayer or few layers). It undergoes transitions from having an indirect to direct band gap, significantly enhancing the probability of light absorption and charge carrier generation at visible wavelengths like 520 nm. The tilt observed in the curves in Figure 3a, with differing directions when the light is switched on and off, can be attributed to trap states and interface effects within the MoTe_2_ material. The decrease in photocurrent when the light is turned on, followed by a slight increase when the light is off, is due to trap states and interface effects within the MoTe_2_ material. When exposed to light, photogenerated carriers quickly accumulate at defect sites, causing an immediate peak. Over time, these carriers recombine or become further captured at trap states, resulting in a slight decrease in photocurrent even though illumination continues. Similarly, upon switching the light off, the trapped carriers are gradually released, leading to a delayed return to baseline and a tilt in the opposite direction.

This behavior reflects the thickness-dependent differences in carrier dynamics between thin and thick MoTe_2_ layers, where the varying densities of trap states and surface characteristics influence the speed and direction of response upon light exposure. Conversely, the photoresponse of thick-based MoTe_2_ is relatively high compared to thin-based MoTe_2_ at 1064 nm, as shown in Figure 3b. This is because thick-based MoTe_2_ has sufficient absorption in the NIR range due to its reduced band gap. The higher performance of the thick MoTe_2_ device in the NIR range, despite its indirect bandgap, is due to increased optical absorption in thicker layers. The additional thickness allows more effective light trapping, enabling greater absorption of photons in the NIR range, which compensates for the indirect band-to-band transitions that are less efficient.

Our Raman spectroscopy results support this explanation: the broader and shifted Raman peaks in the thick MoTe_2_ layer confirm its bulk characteristics and stronger interlayer coupling, which contributes to its indirect bandgap. This structural distinction enhances NIR absorption in thicker layers, allowing for improved photoresponse in this range. Expanding our analysis to measure a broader absorption spectrum for both thick and thin devices would further validate these findings and strengthen the support for the thickness-dependent optical performance of MoTe_2_ devices.

The inconsistency in the current versus time response can be attributed to differences in surface defects, trap states, and carrier dynamics between thin and thick MoTe_2_ layers. Specifically, thin MoTe_2_ layers, with their higher surface-to-volume ratio, tend to have more surface states than trap carriers, resulting in fluctuations in photocurrent. Thicker layers, conversely, exhibit different charge transport properties due to their bulk-like nature, leading to different current behaviors under various light conditions [10]. The rise and decay times are determined by measuring the response times from 10% to 90% and 90% to 10% of the photocurrent when the light source is switched on or off [11]. The rise time constant (*τ*_rise_) values are 0.2 and 0.3 s, while the decay time constant (*τ*_decay_) values are 0.3 and 0.2 s, respectively, for the MoTe_2_ thin and thick-based devices under 520 nm illumination, as demonstrated in Figure 3a. For a comparatively quicker rising time constant of thin-based MoTe_2_ and a decay time constant of thick-based MoTe_2_ heterostructure device, shown in Figure 3a, in a thin-based MoTe_2_ device, the charge carriers exhibit higher speed compared to those in a thick-based MoTe_2_ photodetector. However, this trend is reversed during decay when the thin-based MoTe_2_ structure is exposed to light at 520 nm. The slower response times observed in our MoTe_2_ devices are primarily due to trap states and surface/interface defects within the material. These imperfections capture photogenerated charge carriers, slowing their recombination and transport, which results in delayed photocurrent decay and slower overall response. Additionally, a MoTe_2_-layered structure can contribute to interface effects that impede carrier mobility, further impacting response time. Addressing these limitations through surface passivation techniques, such as an Al_2_O_3_ or h-BN layer, could potentially reduce trap states and enhance response speed by improving carrier mobility and minimizing recombination delays.

Possible degradation by transfer processes might cause a slow decay time. This is due to the dispersive impact resulting from ionic impurities introduced during the transfer procedure and the deterioration induced by the patterning process. Thick-based MoTe_2_ has a significant rise and decay time of 0.1 and 0.2 s, respectively, compared to thin-based MoTe_2_, whose rise time and decay time are 0.1 and 0.3 s, respectively, at 1064 nm, as illustrated in Figure 3b. This demonstrates the superior performance of the thick-based MoTe_2_ device in photodetection, particularly in high-speed applications at 1064 nm.

This is why optimization of the following factors can significantly improve the photoresponse of MoTe_2_ films. First, it has been suggested that the thickness of the MoTe_2_ layers could be optimized to yield improved performance for specific wavelengths. Our study shows that thin MoTe_2_ layers are better for the 520 nm source, and thicker films work better at 1064 nm because of the dissimilarities of successiveness and band structure. The first possible enhancement is that the absorption efficiency for those wavelengths of interest can be maximized by adjusting layer thickness and increasing responsivity and detectivity. The second possible enhancement relates to surface passivation. Defects and cracks at the surface layer for the thin MoTe_2_ films pin the carriers, which in turn results in a decay of photoresponse. A new passivation layer of Al_2_O_3_ or h-BN could reduce the surface defects, thus improving carrier dynamics and, in return, contributing to the increase in photocurrent [12]. Further, doping or alloying MoTe_2_ with elements like Se or S might introduce the means to adjust the bandgap for light absorption and enhance the carrier mobility of the material [13]. This, in turn, would lead to improved photoresponsivity, in particular in certain spectral regions. Another possibility is the enhancement arising from the fusion of MoTe_2_ with other 2D materials in heterostructure architectures. The heterostructures also provide a strong possibility of separating the charges as they form built-in potentials at the interfaces to improve the photocurrent and response time. Furthermore, designing sample structures with plasmonic nanostructures like gold/silver nanoparticles introduces a huge local field enhancement due to plasmonic resonance and improves light absorption photocarrier generation [14]. There is a possibility that the optimization of the metal contacts can significantly enhance the device’s performance. The work function difference and contact resistance of the two metal electrodes can be decreased through a selection of metals with closer work functions or insertion of interfacial layers, leading to a decrease in the Schottky barrier and thus improvement in the photoresponse of the device.

Last, an external gate bias may adjust the carrier density in MoTe_2_ for better charge separation with enhanced photodetector sensitivity under different lighting conditions. From the investigation of the thickness optimization, surface passivation, doping, heterostructure integration, plasmonic enhancement, contact optimization, and external gating, it can be realized that these methods can enhance the photoresponse of MoTe_2_-based photodetectors, and it can thus be more resourceful in sophisticated optoelectronics.

In addition, we carried out essential measurements such as photoresponsivity (*R*), specific detectivity (D*), and *EQE* using the following equations:

(1) $$R=\frac{I_{ph}}{P\times S}$$

(2) $$D^{*}=R\times \sqrt{\frac{S}{2qI_{dark}}}$$

(3) $$EQE=\frac{hcR}{q\lambda }\times 100\%$$

where *I_ph_* represents photocurrent, while *I_dark_* denotes dark current. *P* signifies the incident illumination power density, and *S* represents the illumination area on the channel. The electron charge is denoted by *q*, *h* stands for Planck’s constant, c represents the speed of light, and *λ* indicates the wavelength of incident light [15,16]. At 520 nm, the power density remained 149.64 mW/cm^2^, while at 1064 nm, it was 47 mW/cm^2^, both at a 1 V bias voltage. The thin-based MoTe_2_ exhibited enhanced performance through a wavelength of 520 and thick-based MoTe_2_ at 1064 nm. At 520 nm, it confirmed a responsivity of 1.2 A/W, a detectivity of 4.32 × 10^8^ Jones, and an *EQE* of 285%. In contrast, thick-based MoTe_2_ showed a photoresponsivity of 1.0 A/W, a detectivity of 3.6 × 10^8^ Jones, and an external quantum efficiency of 238% at the same wavelength. At 1064 nm, the thin MoTe_2_ displayed a photoresponsivity of 1.1 A/W, specific detectivity of 3.96 × 10^8^ Jones, and an *EQE* of 127%. For thick MoTe_2_ at this wavelength, the responsivity was 8.8 A/W, specific detectivity was 3.19 × 10^9^ Jones, and *EQE* was 1027%, indicating superior performance compared to thin-based MoTe_2_ at 1064 nm.

We also compared the parameters of our novel thin and thick MoTe_2_ photodetectors with those of traditional photodetection devices, as summarized in Table 1.

**Figure 4:**
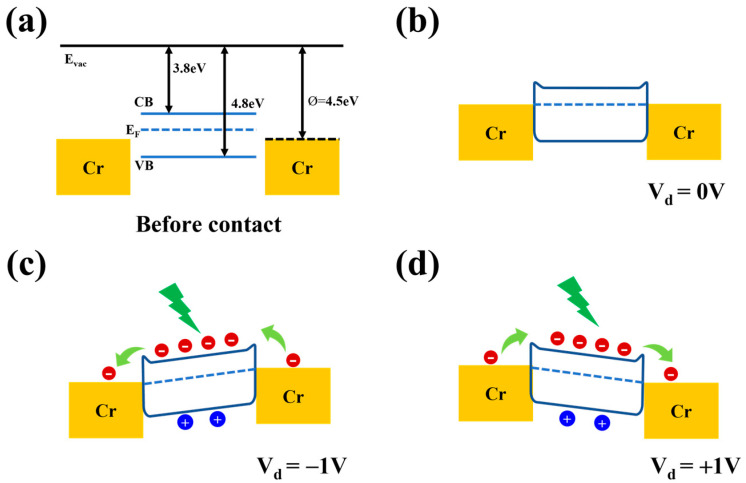
(**a**) Energy band diagrams of MoTe_2_-based photodetectors (*E*_vac_ represents the vacuum energy, and *E*_F_ denotes the Fermi level), with CB indicating the conduction band, VB the valence band, and Ø representing the work function. (**b**) Carrier transfer at *V*_d_ = 0. (**c**) Carrier transfer at applied bias *V*_d_ = −1 V. (**d**) Carrier transfer at *V*_d_ = +1 V.

Moreover, Figure 4 displays the charge transfer mechanism of MoTe_2_-based devices. The MoTe_2_ has diverse Fermi levels with Cr electrodes since Cr electrodes are not in contact with the MoTe_2_ channel, as shown in Figure 4a. The valence band maximum of MoTe_2_ and the work function of Cr have also been determined to be 4.8 eV [32] and 4.5 eV [33], respectively. In the zero-bias condition (Figure 4b), the Fermi level of MoTe_2_ and Cr electrodes is aligned. It remains in equilibrium between the Cr electrodes and the MoTe_2_, with no significant band bending, meaning no photocurrent is generated. Upon exposure to light, by applying *V*_d_ of −1 V, the MoTe_2_ channel absorbs photons, and the electron moves, leaving the holes in the valence band. Fermi level (*E*_F_) shifts towards the valence band of MoTe_2_, creating a substantial energy barrier between the conduction band of MoTe_2_ and the Fermi levels of the Cr electrodes. As a result, the electron pairs generate energy that exceeds the bandgap of the MoTe_2_ flake, producing additional free charge carriers and diminishing the semiconductor’s electrical resistance, as shown in Figure 4c. When applying a +1 V bias voltage, the Fermi level approaches MoTe_2_’s conduction band, forming a minor barrier between MoTe_2_’s conduction band and the Cr electrodes’ Fermi level, as shown in Figure 4d. The application of bias voltage reduces the Schottky barrier height across the junction. The separation of electron-hole pairs results in their rapid movement, where electrons move in opposing directions, and they are gathered by electrodes with Ohmic contact. This process leads to a substantial current (photocurrent) rise between the metal electrodes, effectively traveling to the external circuit and producing a high photocurrent [29,34]. The separation of electron-hole pairs plays a vital role in generating a photocurrent [35,36]. These mechanisms of charge transfer and photo-detection are present across all contacts.

## 4. Conclusions

This study discovered the optoelectronic features of MoTe_2_-based photodetectors by variable thicknesses, precisely relating thin (8 nm) and thick (30 nm) layers. The thin MoTe_2_-based photodetector demonstrated superior responsivity, detectivity, and response time performance compared to thick-based MoTe_2_ at 520 nm. This enhanced performance is attributed to the higher surface-to-volume ratio, better electric field penetration, and improved charge transport properties in the thin layers, facilitating more efficient photogenerated carrier extraction. Conversely, thick-based MoTe_2_ performs better in responsivity, specific detectivity, and EQE at 1064 nm, as described. The enhanced performance is due to the reduced band gap of thick-based MoTe_2_ due to their bulk nature. Both thin and thick MoTe_2_-based devices exhibited n-type behavior. Output characteristic curves specified a photoconductive effect through the thick MoTe_2_ device, showing a more significant increase in drain current under light illumination, highlighting its better responsiveness to fluctuating light power densities. The study concludes that thickness control is crucial in designing MoTe_2_-based photodetectors, with thinner layers at visible and thicker at infrared, providing better sensitivity, faster response times, and improved overall performance. This shows that thickness-dependent MoTe_2_ layers are promising candidates for next-generation photodetection technologies, with applications in imaging, optical communications, and other optoelectronic devices. Future research could explore further optimization through material integration and device structure improvements. In summary, our comparative analysis of thin and thick MoTe_2_-based photodetectors demonstrates the critical impact of thickness on optoelectronic performance. Thin MoTe_2_ layers exhibit superior responsivity in the visible range, while thicker layers excel in the near-infrared region. These findings position MoTe_2_ films as promising candidates for next-generation optoelectronic applications, including visible light photodetectors, infrared detectors, flexible electronics, and high-speed optoelectronic devices. Future work will explore the integration of MoTe_2_ with other 2D materials and device optimizations for specific applications.

## Figures and Tables

**Table 1:** Comparison of photodetectors based on thin and thick MoTe_2_ and two-dimensional material-based heterostructures.

Device	Wavelength (nm)	Responsivity (A/W)	Detectivity (Jones)	E.Q.E	Ref.
AgNPs-MoS_2_	980	8.8 × 10^−4^	1.28 × 10^9^	-	[17]
WSe_2_	780	7.25 × 10^−5^	2.4 × 10^7^	11.55%	[18]
ZnS–MoS_2_	554	1.78 × 10^−5^	-	0.4%	[19]
MoS_2_/PbS	400–1500	4.3 × 10^2^	-	-	[20]
SnS	400–700	4.3 × 10^−3^	71 × 10^6^	-	[21]
MoSe_2_	532	1.7 × 10^−4^	11.58 × 10^8^	0.025%	[16]
SnS_0.25_Se_0.75_	400–700	1.17 × 10^−4^	-	-	[22]
MoS_2_–MoO_3_	405	1.3 × 10^−4^	-	0.041%	[23]
GeP	440	1 × 10^−5^	1.38 × 10^7^	-	[24]
WS_2_	458	2.12 × 10^−6^	-	-	[25]
PbI_2_	450	1.0 × 10^−4^	-	-	[26]
PbI_2_	405	1.3 × 10^−3^	-	-	[27]
ITO/PbI_2_/Au	-	0.5 × 10^−3^	2.5 × 10^12^	-	[28]
MoS_2_	488	4.2 × 10^−4^	-	-	[29]
CdTe	473	1.6 × 10^−4^	5.84 × 10^9^	-	[30]
MoS_2_	514.5	1.1 × 10^−3^	-	-	[31]
MoTe_2_ (thin)	520	1.2	4.32 × 10^8^	285%	This Work
MoTe_2_ (thick)	520	1.0	3.6 × 10^8^	238%	This Work
MoTe_2_ (thin)	1064	1.1	3.96 × 10^8^	127%	This Work
MoTe_2_ (thick)	1064	8.8	3.19 × 10^9^	1027%	This Work

## Data Availability

Specific features of the study and the original findings are described in the article; if you have any other questions, they should be addressed to the corresponding author.
